# Exploiting the Fc base of IgG antibodies to create functional nanoparticle conjugates

**DOI:** 10.1038/s41598-024-65822-7

**Published:** 2024-06-27

**Authors:** Mohammed M. Al Qaraghuli, Karina Kubiak-Ossowska, Valerie A. Ferro, Paul A. Mulheran

**Affiliations:** 1 EPSRC Future Manufacturing Research Hub for Continuous Manufacturing and Advanced Crystallisation, University of Strathclyde, Glasgow, UK; 2 SiMologics Ltd. The Enterprise Hub, Level 6 Graham Hills Building, 50 Richmond Street, Glasgow, G1 1XP UK; 3 Department of Chemical and Process Engineering, University of Strathclyde, 75 Montrose Street, Glasgow, G1 1XJ UK; 4 Archie-West, Department of Physics, University of Strathclyde, 107 Rottenrow East, Glasgow, G4 0NG UK; 5 Strathclyde Institute of Pharmacy and Biomedical Sciences, University of Strathclyde, 161 Cathedral Street, Glasgow, G4 0RE UK

**Keywords:** Antibody, Gold nanoparticles, Structural similarity, Antibody function, Molecular dynamics, Antibody therapy, Biosensors, Nanoparticles, Diagnostic devices, Nanotechnology in cancer, Computational nanotechnology, Computational models, Protein design

## Abstract

The structures of the Fc base of various IgG antibodies have been examined with a view to understanding how this region can be used to conjugate IgG to nanoparticles. The base structure is found to be largely consistent across a range of species and subtypes, comprising a hydrophobic region surrounded by hydrophilic residues, some of which are charged at physiological conditions. In addition, atomistic Molecular Dynamics simulations were performed to explore how model nanoparticles interact with the base using neutral and negatively charged gold nanoparticles. Both types of nanoparticle interacted readily with the base, leading to an adaptation of the antibody base surface to enhance the interactions. Furthermore, these interactions left the rest of the domain at the base of the Fc region structurally intact. This implies that coupling nanoparticles to the base of an IgG molecule is both feasible and desirable, since it leaves the antibody free to interact with its surroundings so that antigen-binding functionality can be retained. These results will therefore help guide future attempts to develop new nanotechnologies that exploit the unique properties of both antibodies and nanoparticles.

## Introduction

Nanotechnology is crucial in both therapeutic and diagnostic applications, as it can be used for targeted drug delivery and precise imaging, offering a personalised and efficient approach to medicine. Nanoparticles demonstrate key physicochemical properties such as surface topology/morphology, controllable aggregation, and high surface area-to-volume ratio^1^. Various types of nanoparticles have been utilised, including graphene, gold, iron oxide, silica, and organic materials^2^. Of these, gold nanoparticles (AuNPs) demonstrate high potential as they possess novel physico-chemical properties and have a good safety profile^3^. These nanoparticles could be conjugated to biomolecules called antibodies to create a synergistic platform with diverse applications. Optimising the interactions between antibodies and nanoparticles is essential to enhance binding affinity, reduce off-target effects, and maximise the sensitivity of diagnostic tests. This fine-tuning not only enhances therapeutic efficacy, safety, and stability but also improves ongoing research, and pushes the boundaries of medical innovation.

In this work, we have investigated how antibodies (specifically IgG) interact with nanoparticles in a way that avoids hindering the various functional regions on the antibody. By understanding this mechanism in detail, we reveal universal features that can be exploited in future nanotechnological designs of functional conjugates. This is timely, since monoclonal antibodies (mAbs), targeting a specific biomarker, are transforming the pharmaceutical industry through major contributions to both therapeutics and diagnostics. The U.S. Food and Drug Administration (FDA) approved sixteen antibodies by November 2023^4^. The potential to exploit these advances further through controlled interaction with nanoparticles, which bring their own additional functionality, is therefore significant, but hinges on our understanding of the process fundamentals.

**Figure 1:**
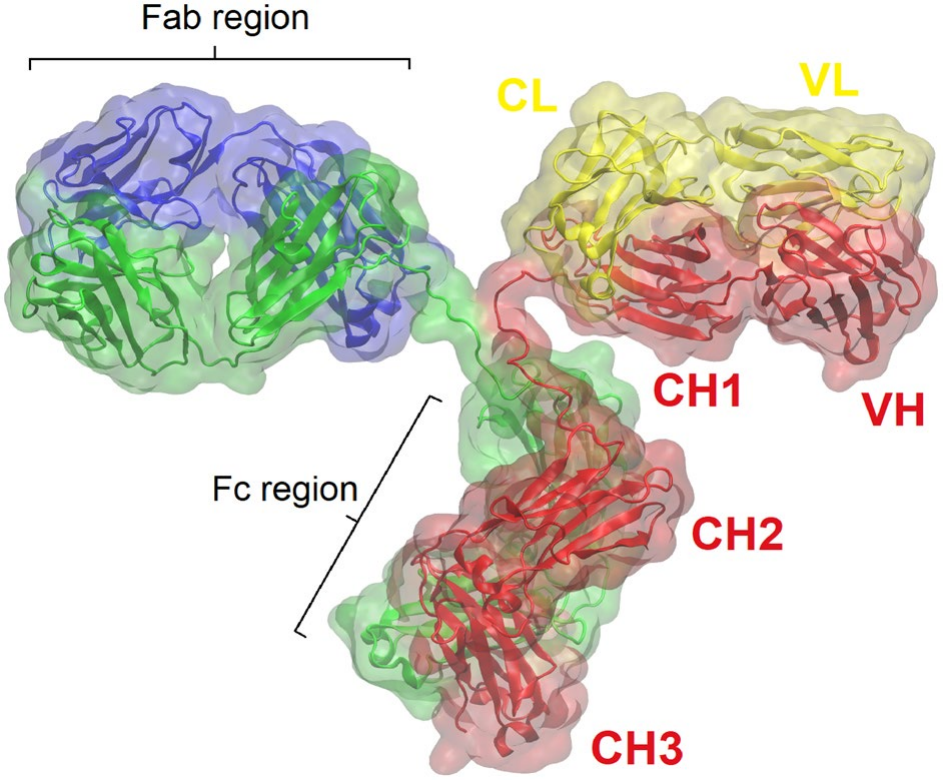
IgG Antibody structure exemplified by the Protein Data Bank entry 1IGT, viewed in VMD^9^.

It is useful to review in broad terms the structure of antibodies, which are glycoproteins expressed as five different antibody isotypes, known as IgG (~ 80%), IgA (~ 10%), IgM (~ 5%), IgD (< 1%) and IgE (< 1%); out of the four human IgG subclasses, isotypes IgG_1_ and IgG_4_ are most used for therapeutic purposes as mAbs^5^. An IgG antibody is composed of two heavy and two light chains that are held together by disulphide bonds (see Fig. 1). These four chains are arranged to form two Fragment antigen binding (Fab) regions and one Fragment crystallisable (Fc) region^6,7^. Each heavy chain of the IgG, IgA, and IgD isotypes folds into four domains: one variable (V_H_), and three constant domains (C_H1–3_). Each light chain is composed of one variable (V_L_) and one constant (C_L_) domain, forming the Fab region along with the heavy chain V_H_ and C_H1_ domains. The antigen recognition process is accomplished by the complementarity determining regions (CDRs) located at the tips of the Fabs, occasionally being supported by a few residues from the framework regions (highly conserved regions that act as a scaffold for the CDRs)^8^. MAbs normally undergo refinement and affinity maturation processes to specifically bind to their target site (the epitope) using the surface created by these CDRs (the paratope).

While the Fab fragment is responsible for antigen recognition through its variable sites, the Fc region can propagate a series of effector responses, including antibody-dependent cellular cytotoxicity (ADCC) and complement-dependent cytotoxicity (CDC)^10,11^. The amino acid positions in the C_H2_ domain that are in proximity to the hinge region (where the Fabs join the Fc, see Fig. 1) are responsible for effector functions of antibodies as they contain largely overlapping binding sites for C1q (the antibody-antigen complex binding complement) and IgG-Fc receptors (FcγR) on innate immune system effector cells^11^. The interface between the two C_H2_/C_H3_ domains contain additional important sites for glycosylation (position 297)^12^. This interface also contains the binding site for the neonatal Fc receptor (FcRn), which is responsible for IgG placental passage, prolonged half-life, and transport across mucosal surfaces^13^.

Given the important roles played by these various regions on the antibody, it is clear that interactions with nanoparticles could compromise various antibody functions. Fortunately, one crucial region at the antibody base (which is suitably distant from the functional regions) is also available for nanoparticle interactions, and furthermore it appears to have some universal features across various antibody types, making it an important region for further study.

The C_H3_ domains of the two heavy chains are arranged as a cross at the C-terminus of the antibody (see Fig. 1), and this is the area that will be referred to as “the antibody base” in this paper. Biotechnology companies like F-star Therapeutics (https://f-star.com/) have targeted this region in their antibody-based technology to enable the creation of two additional distinct antigen binding sites in the Fc region of a natural antibody, termed an Fcab. The antibody base could also be exploited for conjugation of antibodies to various nanoparticles, and the generated conjugates could then be used for technological applications.

Here, we have analysed the antibody base in terms of surface topography, amino acid composition across different classes, and surface charge. In addition, the ability of this base to be conjugated to model AuNPs that are either neutral or negatively charged was also investigated using Molecular Dynamics (MD) simulations. These analyses provide a solid foundation that improves our understanding of this important region of an IgG antibody molecule and paves the way for the development of efficient conjugates that can be used in diagnostic or therapeutic applications.

## Results and discussion

### Main chain conformation and sequence analyses

**Figure 2:**
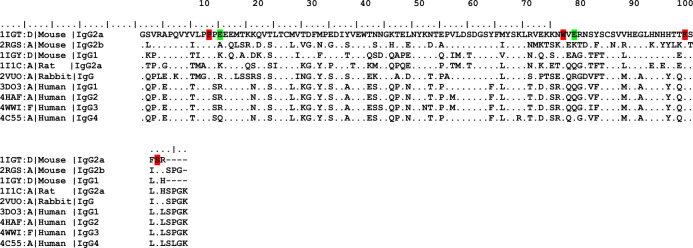
Sequence alignment of the C_H3_ domains. Amino acids similar to each relevant position of the first structure (1IGT) are denoted as points. The four conserved positions that contribute to the base are highlighted in red, whilst the two most variable positions are coloured in green. The sequences were aligned using BioEdit Sequence Alignment Editor, version 7.2.5^14^.

Fifty-four structures were retrieved from the Protein Data Bank (PDB) representing different IgG subclasses: human IgG_1_ (38 structures), human IgG_2_ (3), human IgG_3_ (2), human IgG_4_ (5), rabbit IgG (1), mouse IgG_1_ (2), mouse IgG_2a_ (1), mouse IgG_2b_ (1), and rat IgG_2a_ (1). One of these from each distinct species/subclasses were selected as representatives, giving nine structures for detailed analysis. Alignment of the respective C_H3_ domains revealed differences in these sequences at various positions of the different species, however, these variances were less noticeable across the same species, as can be seen in Fig. 2.

**Figure 3:**
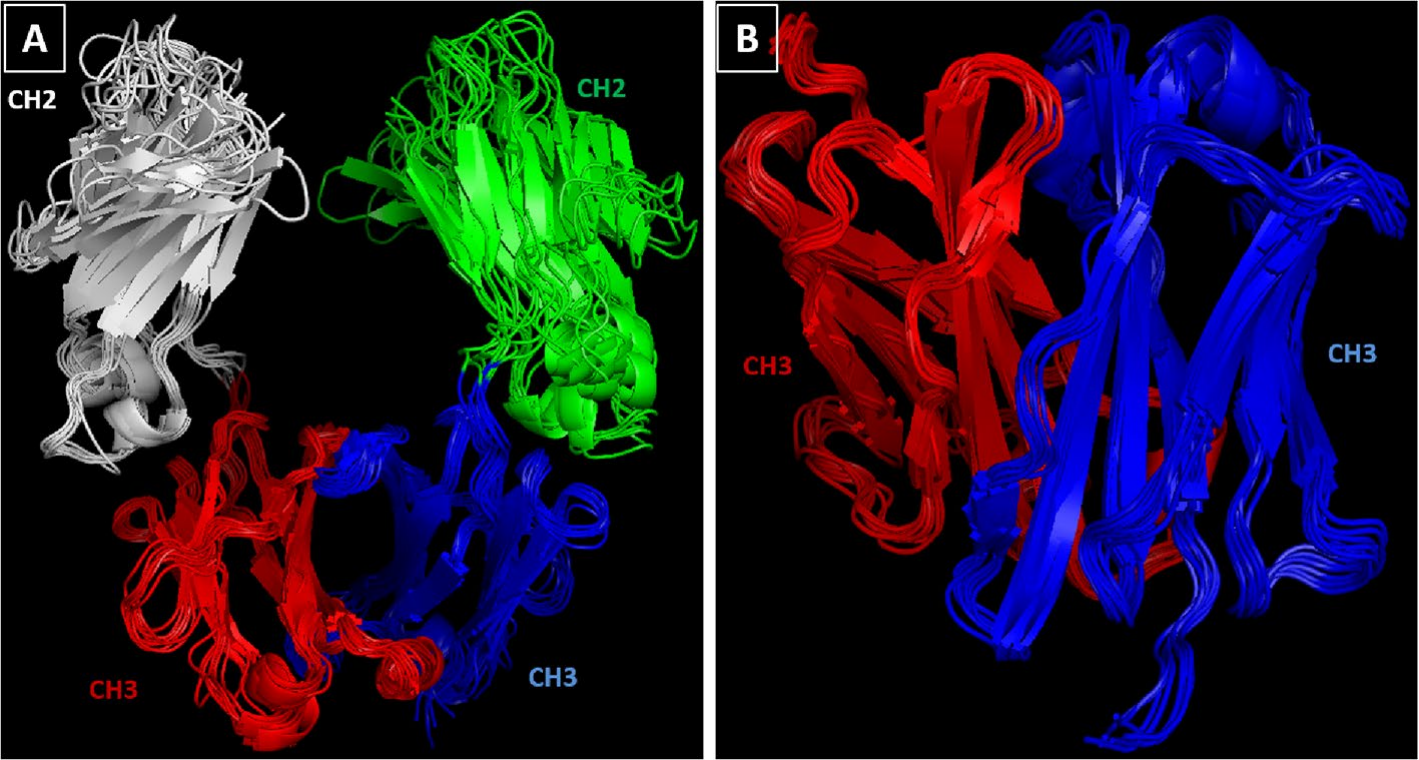
Comparison of the structural configurations of the Fc regions. The C_H2_ and C_H3_ domains of the Fc region (**A**), and the C_H3_ domains alone (**B**) aligned and viewed with PyMOL software.

Despite the sequence difference, Fig. 3 shows that C_H3_ domains have very similar structural configurations, even greater than the C_H2_ domains of the same antibodies, although these too show common structural motifs. The two C_H3_ domains (one from each heavy chain) pack firmly with each other in contrast to the C_H2_ domains that have no observable protein–protein contacts with one another, but instead fill the space between them with highly conserved N-glycan (not shown in Fig. 3) attached at Asn297^15^.

The antibody base has previously been analysed^16^, revealing that most of the 19 residues in the IgG C_H3_ β-strand interface are homologous or identical. Table 1 shows that sequence variances were evident in 15 out of the 19 positions in different antibody classes and species. The four conserved positions were observed at positions P354, W418, K440, and S443 (we have used the pdb numbering throughput this paper) as shown in Table 1 and highlighted in red in Fig. 2. This conservation might be attributable to the aforementioned positions, as well as the interface configuration between two C_H3_ domains, being intolerant to mutation^17^. For instance, P354 demonstrates remarkable importance for biophysical properties, since isomerisation from trans- to cis-proline was shown to be the rate‐limiting step in C_H3_ folding^18^. Furthermore, the same authors proved that prolyl isomerisation at this position is necessary for C_H3_ homodimer formation.

**Table 1:** Amino acids that construct the Fc base. A total of 19 amino acid positions (in each heavy chain) forms the base of the Fc region. Each amino acid, within the analysed sequences, was numbered according to the Kabat scheme^8,19^ as well as the corresponding number in the PDB files. The four conserved positions are highlighted in bold.

	Amino acids	Position number (Kabat)	Number in PDB files
1	V/I/T	371	351
2	L/I/M	372	352
3	P/A/G	373	353
**4**	**P**	**374**	**354**
5	P/S	375	355
6	E/A/K/R/Q	376	356
7	E/Q	377	357
8	M/L	381	359
9	T/S/A	382	360
10	K/T	445	415
11	K/S/E	446	416
**12**	**W**	**448**	**418**
13	V/E/Q	449	419
14	E/K/A/Q/R	450	420
15	R/T/G	451	421
**16**	**K**	**470**	**440**
17	F/I/L	472	442
**18**	**S**	**473**	**443**
19	R/H/L	474	444

### Surface structure and charge at the base of the Fc region

**Figure 4:**
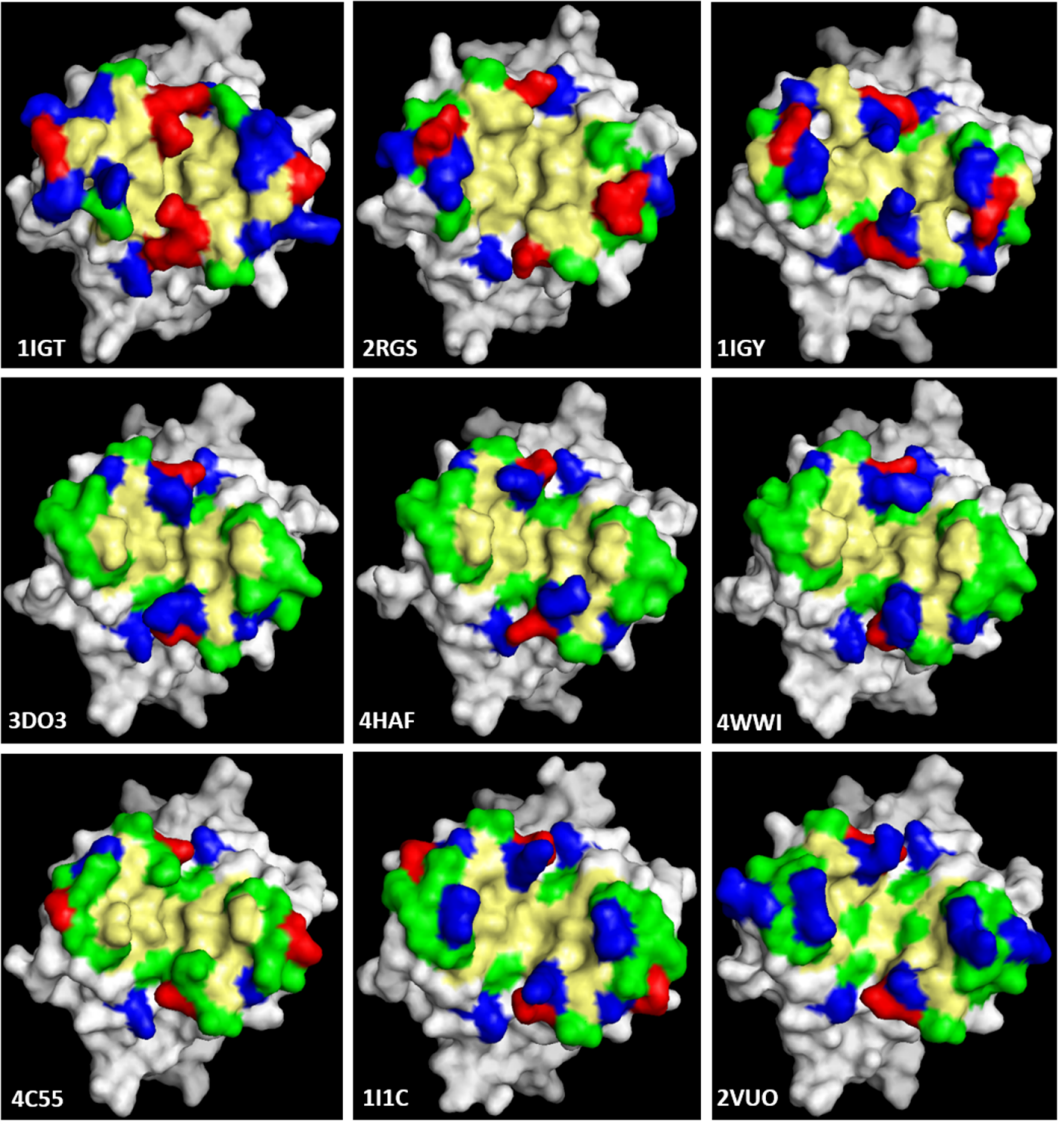
Amino acid distribution at the Fc base. The amino acids are coloured by type: yellow for hydrophobic [Ala (A), Val (V), Leu (L), Ile (I), Pro (P), Met (M), Phe (F), and Trp (W)], green for hydrophilic [Gly (G), Ser (S), Cys (C), Asn (N), Gln (Q), and Tyr (T), blue for positively charged [Lys (K), Arg (R), and His (H)], and red for negative [Glu (E) and Asp (D)]. PDB entries are depicted at the lower corner of each image. Structures were imaged with PyMOL.

The base of the Fc region is commonly characterised by the presence of hydrophobic amino acids in the central region (positions 351, 352, 353, 354, 355, and 359), which are surrounded by hydrophilic and charged amino acids (see Fig. 4). This appears to be a general structural trend, although some exceptions or modifications in this general pattern were observed. In the two strands of the outer β-sheet (strands C and F), alternate residues are found to be tolerant and intolerant to mutation^17,20^. This has been attributed to the orientation of the residues in a β-sheet, by which the side chains of the hydrophobic residues, as well as the disulphide bond, are all directed to the hydrophobic core of the C_H3_ domain. In addition, the same authors suggested that intolerance to mutation of a particular residue is due to side‐chain interactions with other parts of the molecule, and not primarily caused by its location in the β-sheet.

Major differences are observed between mouse and human domains at positions 415, 416, 420, and 421. These positions are occupied by charged Lys (K) and Glu (E) in mouse antibodies, whilst hydrophilic Thr (T), Ser (S), Gln (Q), and Gly (G) dominate these positions in human antibodies (Fig. 2). The rat and rabbit domains are highly similar to their human counterparts except in the presence of Arg (position 420) and Glu (position 416) in rabbit and rat domains, respectively.

The amino acid architecture of the selected antibody bases is reflected in their surface-mapped electrostatic potential (Supplementary Fig. 1). The human IgG_4_ base (4C55) displays negatively charged patches, which can be attributed to the presence of Glu at position 420, and the absence of positively charged amino acids at position 356; note also the low isoelectric point (PI) of this domain (Supplementary Tab. S1 and Fig. S2). At the same time, the central region of the remaining three human isotypes (3DO3, 4HAF, and 4WWI) are neutral, which can be linked to their slightly higher PI and the presence of Arg and Gln at positions 356 and 420, respectively (Supplementary Tab. S1 and Fig. S2). The remaining five structures, from mouse, rabbit, and rat, are characterised by a mix of neutral, positive, and negative charges (Supplementary Tab S1 and Fig. S2).

From the picture emerging from the residue types and their charge, it appears that the hydrophilic/hydrophobic pattern will be more important for recognising a substrate (on which the Fc could attach) than the electrostatic one, which might only play a secondary role in any recognition process.

### Molecular dynamics

Our analyses include three scenarios using the 1IGT antibody as an exemplar of the Fc base structure. We note that a structural analysis of the 1IGT antibody has been performed previously using molecular simulations^21^. Firstly, the antibody was simulated in isolation in a box of explicit water and ions. Secondly, a negatively charged AuNP with diameter 2.5 nm was included in the box, so that interactions with the antibody naturally developed. Finally, an uncharged AuNP with the same size was used. We performed two independent trajectories for each of these systems, and since these were broadly similar in behaviour in each case, we analyse in detail the results from one of each.

**Figure 5:**
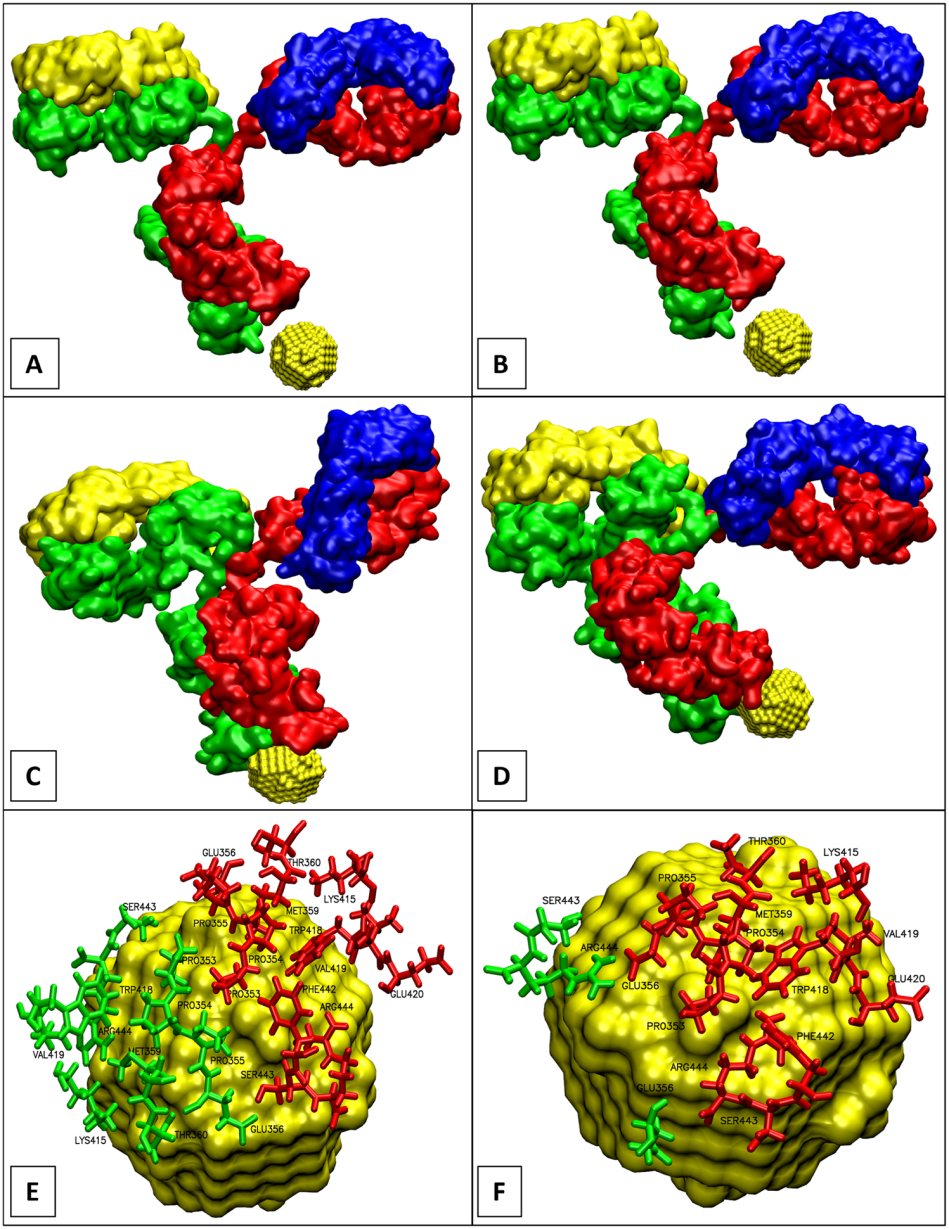
Simulation images of the 1IGT antibody interacting with the AuNPs. Binding of the 1IGT antibody to the negatively charged AuNP, before (**A**) and at the end of a 100ns trajectory (**C**). Binding of 1IGT to the uncharged AuNP, before (**B**) and at the end of a 100ns trajectory (**D**). The amino acids involved in the binding process (within 5Å from the AuNP at the end of the 100ns trajectories) are labelled for charged (**E**) and uncharged (**F**) AuNPs. The four chains of the antibody are coloured separately as follows: blue light chain A, red heavy chain B, yellow light chain C, green heavy chain D.

The binding dynamics of both model AuNPs, in addition to the unconjugated antibody, can be observed in Supplementary videos V1–V3. Images from the interaction of the Fc base with the nanoparticles are provided in Fig. 5. The 1IGT antibody in all three simulations was stable. In addition, both the charged and uncharged AuNPs started to interact with the amino acids at the base as early as the minimisation stage of the simulations. Some amino acid side chains were within 5—7 Å of the AuNPs after approximately 1 ns and 2 ns for the charged and uncharged simulations, respectively. Such rapid interactions are unusual for interactions not dominated by electrostatics^22^, which suggests a perfect “architecture” of the base which allows for rapid recognition and adsorption to the substrate. Both heavy chains of the 1IGT antibody contributed strongly to the binding to the charged AuNP (Fig. 5E). However, one of the heavy chains (chain B, red in Fig. 5F) interacted with the uncharged AuNP more than the other (chain D, green in Fig. 5F).

Thirteen amino acids from heavy chain B contributed to the binding to the charged and uncharged AuNPs, as listed in Table 2, while heavy chain D contributed eleven and three amino acids, respectively. The contribution of the amino acids reflects the surface charge of the AuNPs. The difference between the interactions with the charged and uncharged AuNPs occurred only in heavy chain D where the list of involved residues is much shorter and consists only hydrophilic residues. It suggests that, as expected, the lack of AuNP charge causes the interactions to be slightly weaker and hydrophilic effects are more visible, while in the case of charged AuNP the interaction is dominated by electrostatics and therefore more subtle effects such as hydrophilic interactions are slightly hidden hence more difficult to detect. Nevertheless, careful inspection of the residues listed in Table 2 indicates that both effects play an important part in AuNP-antibody interactions.

**Table 2:** Amino acids involved in the binding to the AuNPs**.** The amino acids involved in the binding process (within 5 Å from the AuNP at the end of the 100 ns trajectories) are listed for each of the heavy chains B and D. Hydrophilic amino acids might be positively charged (+), negatively charged (−) or neutral (n) at pH7 as indicated in the table.

Nature of amino acids	Charged NP		Uncharged NP	
	Chain B	Chain D	Chain B	Chain D
Hydrophobic	Pro 353	Pro 353	Pro 353	–
Hydrophobic	Pro 354	Pro 354	Pro 354	–
Hydrophobic	Pro 355	Pro 355	Pro 355	–
Hydrophilic (−)	Glu 356	Glu 356	Glu 356	Glu 356
Hydrophobic	Met 359	Met 359	Met 359	–
Hydrophilic	Thr 360	Thr 360	Thr 360	–
Hydrophilic (+)	Lys 415	Lys 415	Lys 415	–
Hydrophobic	Trp 418	Trp 418	Trp 418	–
Hydrophobic	Val 419	Val 419	Val 419	–
Hydrophilic (−)	Glu 420	–	Glu 420	–
Hydrophobic	Phe 442	–	Phe 442	–
Hydrophilic	Ser 443	Ser 443	Ser 443	Ser 443
Hydrophilic (+)	Arg 444	Arg 444	Arg 444	Arg 444

**Figure 6:**
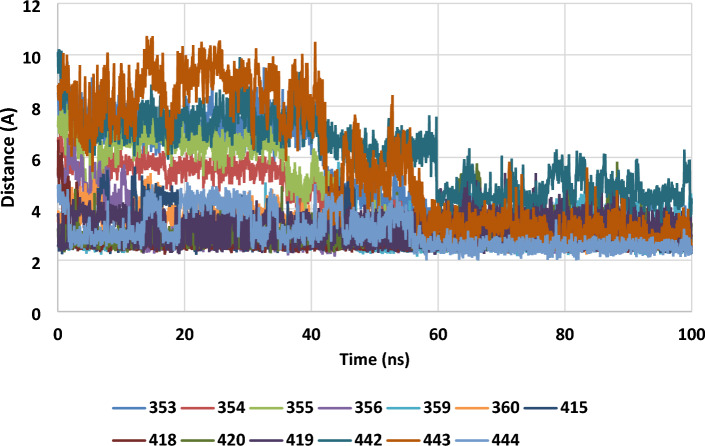
The time evolution of the minimum separations (in Å) between the interacting residues of the I1GT Chain B and the charged AuNP. The residue numbers are shown in the legend.

In Fig. 6, the minimum separations between the the thirteen residues of Chain B listed in Table 2 and the charged AuNP are plotted over the course of the 100 ns trajectory. While not all the data are distinguishable at all times, the general trends are clear, and after about 60 ns all the residues stay within ~ 4 Å of the nanoparticle, except for Phe 442 which stays within 6 Å. This shows that the interactions are long-lived, and that these residues have reduced mobility once they interact with the nanoparticle. Similar results for Chain D interacting with the charged AuNP, and for chains B and D interacting with the uncharged AuNP, are shown in the supplementary Figure S3.

**Figure 7:**
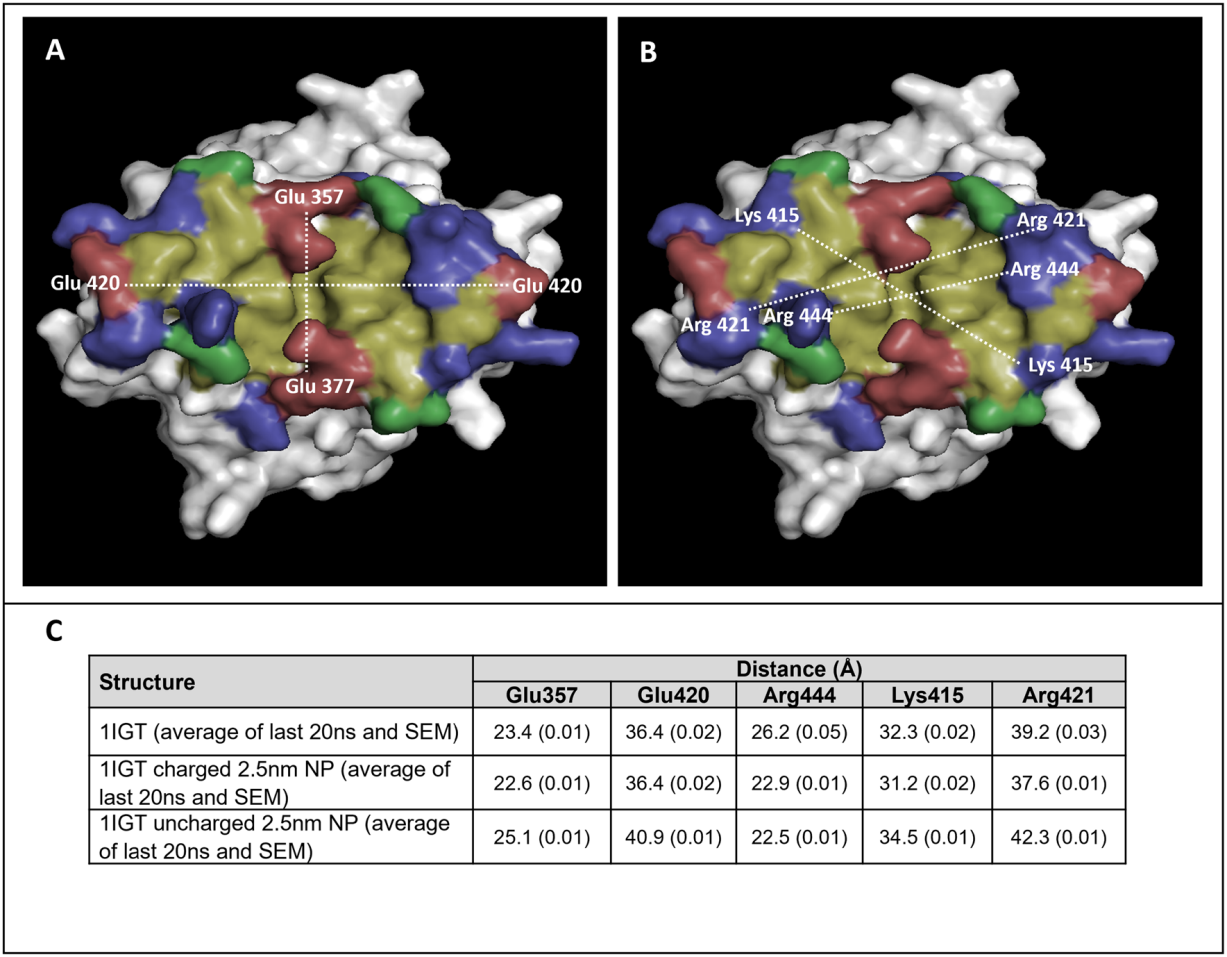
Fc base surface measurements. (**A**) Location of negatively charged positions on the Fc base. Two Glu positions (377 and 420) were selected in the two C_H3_ chains that form the antibody base. (**B**) Location of positively charged positions. Three positive positions (Lys 415, Arg 421, and Arg 444) were selected in the two C_H3_ chains. (**C**) The distances between these positions (specifically the α-carbon) in the two C_H3_ domains are presented in the table. In addition, the average distances were also measured throughout the last 20 ns of the simulation, and the standard error of the means (SEM) are denoted in brackets.

To monitor the changes in the Fc base structure caused by interactions with the AuNPs, the separations of two negatively charged amino acids (377 and 420) and three positively charged residues (415, 421, and 444) were tracked by measuring the distance between them in the two chains in each simulation (see Fig. 7). The distances measured revealed that in the uncharged AuNP simulation, larger surface area is preferred to expose more hydrophobic positions in the Fc base centre. In contrast, interactions with the negatively charged AuNP favours a smaller surface area, with the positively charged residues tending to be drawn in towards the nanoparticle.

**Figure 8:**
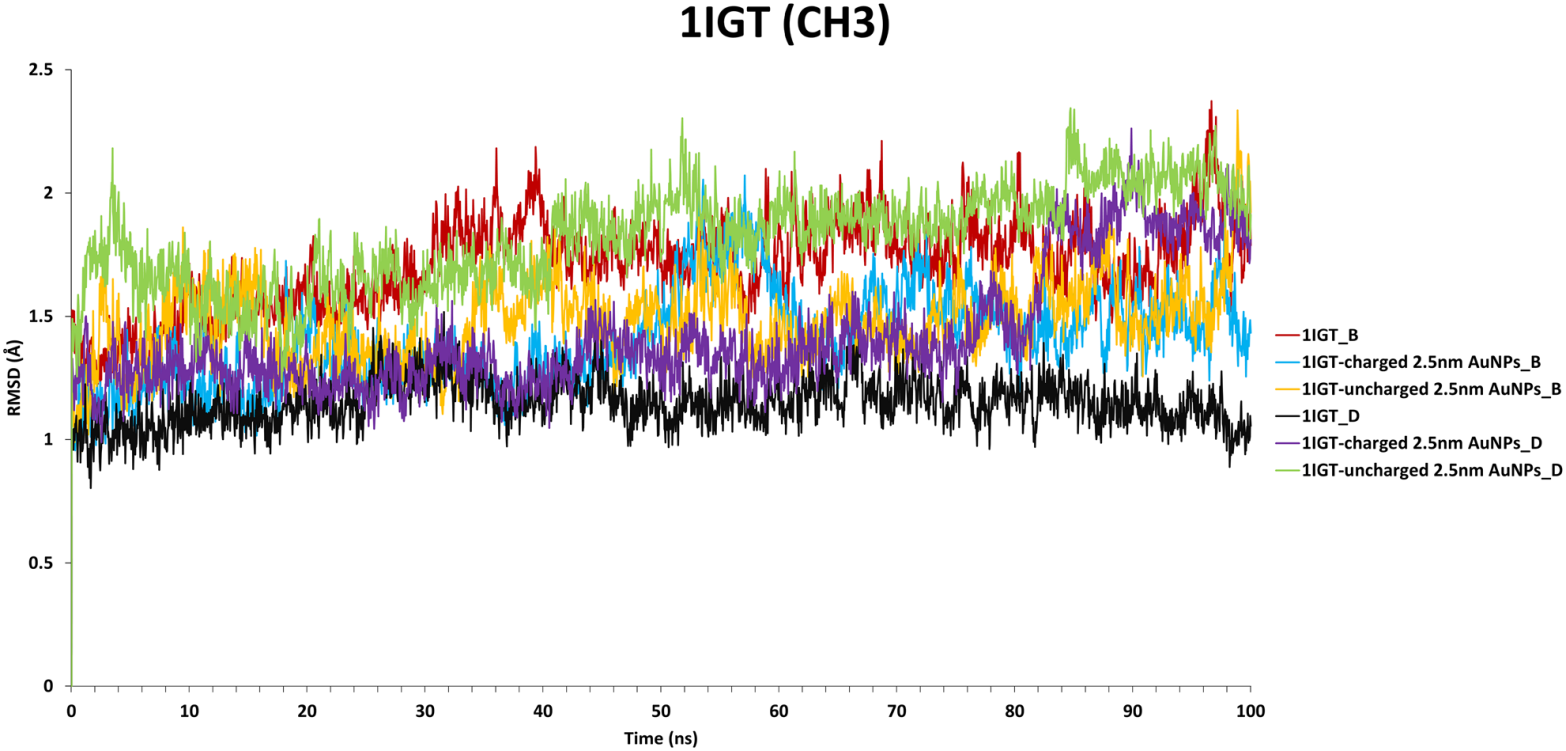
The RMSD of the C_H3_ domains for the three simulations. Statistical analyses are presented in Table S2.

It was essential to test the effect of AuNP interactions on the structure of the 1IGT antibody to see if binding destabilised it; this could have significant consequences for conjugate design. The RMSD, as defined in the Methods Section, of both the heavy chain (B and D) C_H3_ domains for each of the three analysed simulations (1IGT antibody alone or conjugated to charged or uncharged AuNPs) were measured through the course of the trajectories (Fig. 8). It can be seen that in all cases the RMSD values plateau in the range 0.8–2.3 Å, which indicates structural stability of the C_H3_ domains, which are over an order of magnitude larger in size.

In general, the RMSD values of the heavy chain B were higher than that of heavy chain D in the free 1IGT antibody (Fig. 8; Tab. S2). In the charged and uncharged simulations, chain B exhibited reduced conformational flexibility compared to the free antibody simulation. This is probably due to the participation of 13 amino acids from this chain in the interaction with the charged and uncharged AuNPs (Table 2). As noted earlier, heavy chain D has a low contribution to the binding to uncharged AuNP (Tab. 2 and Fig. 5F), and thus can show higher conformational flexibility throughout the simulation.

RMSF analysis was additionally performed (Supplementary Fig. S4) for the chains in the various simulations, using the final 20 ns of each trajectory. They indicate that the RMSF values remain consistent across the simulations, indicating again the good structural integrity of the C_H3_ domains even when interacting with the nanoparticles at the base. Our previous experience with the same antibody indicates that an RMSF of 5 Å for a residue in an α-helical structure is considerable and normally indicates that the helix structure is strongly affected or even unfolded; while the same value is modest for a residue in a loop region or residue responsible for binding a ligand^21^. The RMSF values shown in Fig. S4 are all consistently below 5 Å.

## Summary and conclusions

Our analysis of the base of representative IgGs shows that there is potential for exploitation in future nanotechnologies. Providing that it can be targeted, the base is an ideal location for conjugation, since it leaves the rest of the IgG structures free to interact with the environment. Importantly, we have found a common structural motif that suggests a generality in the conclusions we have drawn. This motif has a hydrophobic region surrounded by hydrophilic residues, some of which are charged at physiological conditions.

We have also used atomistic MD to study how model nanoparticles interact with the base motif in an exemplar IgG. The AuNPs were either neutral or negatively charged. In both cases, we found that the nanoparticles interact strongly with the IgG base, where the hydrophobic region facilitates interaction with the neutral nanoparticle while the charged residues surrounding the region facilitate interactions with the charged AuNP. In both cases, the structure of the IgG domains that contribute to the base show good stability, suggesting that the antibody retains its functionality despite the nanoparticle interactions.

Whilst we have used model AuNPs, the generality of the base structures and nature of the resulting interactions suggest that the results will be transferrable to other IgG-nanoparticle conjugates. Nanoparticles in solution tend to be negatively charged, and the interactions with the neutral AuNP provides insight into how the IgG might interact with neutral nanostructured surfaces. In both cases, there is cause for optimism that conjugation at the base is feasible, so that in a self-assembled scenario, where IgG in solution is mixed with nanoparticles or exposed to a nanostructured substrate, some functional conjugates will naturally form. Subsequently, the specific results of this study can help guide the development of future AuNP-based technologies.

Additional future work could be directed towards analysing the involvement of the specified residues in nanoparticle interactions by examining the binding free energy for the interaction as proposed by other researchers^23^.

## Methods

### Fc structure selection

The structures of various IgG antibodies were retrieved from the PDB using the following search terms: "monoclonal antibody", “immunoglobulin”, “immunoglobulin G”, "antibody Fc region", or "antibody”. Only structures with a resolution of 3 Å or lower were included in the analysis to allow a confident determination of the molecular interactions and structures^24–26^. Using this search profile, 54 Fc crystal structures of different IgG subclasses were identified. Another 33 human Fc structures were additionally identified, but not included in this analysis, because they were either heterodimers or mutated domains of bispecific antibodies.

### Sequence analysis and electrostatic potential

Sequences of the selected structures were aligned using ClustalW Multiple alignment of the BioEdit Sequence Alignment Editor, version 7.2.5^27^, and the full sequences are listed in the Supplementary Material Fig. S1. Two molecular visualisation software packages, PyMOL (The PyMOL Molecular Graphics System, Version 1.7.4 Schrödinger, LLC.) and Visual Molecular Dynamics (VMD 1.9.1)^9^, were used to visualise and analyse the crystal structures. The molecular weight (Mwt) and isoelectric points (PI) of the selected structures were calculated using ExPASy Bioinformatics Resource Portal^28^. The electrostatic potential of the selected structures were calculated using Python Molecule Viewer (PMV) Version 1.5.6^29^ using the Adaptive Poisson-Boltzmann Solver (APBS) Version 0.5.1. The energy was mapped to the surface with medium surface quality and at 1Å distance from the surface (Compute > Electrostatics > Map Potential to Surface).

### Molecular simulations

All simulations were executed with the NAMD 2.11 programme^30^, using the CHARMM27 force-field, and the results were evaluated using VMD 1.9.1 software. As reported by Martin-Garcia et al.^31^*,* the chosen force field guarantees reliable simulations with results in reasonably good agreement with experimental data. An IgG_2a_ mouse antibody crystal structure (PDB ID: 1IGT), which will be denoted as the 1IGT antibody throughout, was selected as a representative exemplar model^32^. The selection of the 1IGT structure was based on several critical factors. First, 1IGT is a mouse IgG2a antibody, which is a functional equivalent to human IgG1 antibodies, commonly used in therapeutic applications^33,34^. This makes it a valuable model for understanding therapeutic antibodies. Second, 1IGT is among the most extensively studied crystal structures in antibody research, which enables comprehensive comparisons with other studies. Furthermore, unlike other reported human crystal structures (3DO3, 4HAF, 4WWI, and 4C55), which only include the Fc region, 1IGT reports a complete antibody structure. This provides a more accurate representation of antibody behaviour, including mechanisms such as allosteric movements and the influence of the Fab regions. Therefore, 1IGT is an appropriate model that provides a reliable basis for understanding the adsorption process.

Both uncharged and charged spherical gold nanoparticles (AuNPs), diameter 2.5 nm, were independently used as adsorption models in all systems. The size selection of AuNPs was directed by the size of the antibody base to allow a meaningful analysis of the antibody-NP interaction. The chosen size allowed the AuNP to fit snugly into the antibody base. Selecting larger nanoparticles or a flat gold surface might have altered the surface topography of the antibody base, and could potentially result in a flatter surface. This change could mimic the response of antibody binding sites when interacting with large protein targets^35^. However, this aspect requires further investigation and validation in future studies.

The initial atomic coordinates for the nanoparticles were generated from a bulk face-centered cubic (fcc) crystal structure, and the well tested interatomic potential developed by Heinz et al.^36^ was used in the simulations to create the equilibrated structure. This force field has been used successfully with neutral solvents, and has been recently used to study biopolymers^37^, organic molecules^38,39^, protein^40–42^ and peptide^43,44^ interactions with AuNPs. It has also been incorporated into newly developed software packages^45^. The model charged AuNP was identical to the uncharged one, except a charge of − 0.05 e was associated with each surface atom in the nanoparticle, giving it a total charge of − 10 e. It is important to emphasise that in this instance we report the list of residues interacting with the AuNP, which are found based on a distance threshold of 5–7 Å. The precise orientation of particular residues’ side chains was not analysed because of (i) the overall system mobility, (ii) the antibody flexibility allowing it to locally adopt suitable adsorption to the AuNP, and (iii) variations in antibody sequences. These factors could potentially influence the exact orientation of residue side chains during the adsorption process. Therefore, a detailed analysis of side-chain orientation is not included, as different antibodies may utilise various specific residues and mechanisms to facilitate adsorption. Instead, here we focus on the common structural motif of the Fc bases (namely a hydrophobic patch surrounded by hydrophilic residues) and how this facilitates interaction with the nanoparticles.

An initial simulation was performed to find the equilibrium structure of the AuNP in water only, and to confirm its stability. The AuNP system was firstly solvated in a cubic water box (TIP3P model) that was extended at least 30 Å from any AuNP atom. Then NaCl salt with ionic strength I = 5 × 10^–2^ M was added (15 Na^+^ and 15 Cl^-^ ions in the case of a neutral AuNP). In general, the simulation was conducted in three stages, starting with water and ion minimisation (100 ps; integration step 1 fs), and followed by minimisation of the entire system (water, ions, and AuNP) for 10,000 steps, and then equilibration at 300 K for 6 ns with integration step 2 fs. The final production simulation was simulated for 10 ns with a 2 fs time step. The AuNP atoms were immobilised only during the initial minimisation stage. The simulations were performed under the NVT ensemble with a Langevin thermostat as implemented in NAMD. The cutoff for VdW interactions was 12 Å while for the electrostatic part of the nonbonding interactions the PME method was used^46^. To reduce the computational time, water molecules were treated as rigid bodies (using the RATTLE algorithm).

The 1IGT-AuNP systems were prepared by positioning the charged or uncharged AuNPs close to the base site of the “Y” shaped 1IGT antibody. The AuNPs were placed 10 Å away from the antibody (distance measured between the surfaces) to avoid biased adsorption, reduce the time required for free diffusion, and guide the adsorption to the desired region of the antibody. The 1IGT-AuNP systems were initially solvated in a rectangular water box which extended at least 30 Å from any antibody or AuNP atom and again NaCl was added at an ionic strength I = 5 × 10^–2^ M (213 Na^+^ and 213 Cl^-^ ions were introduced with the neutral AuNP). The size of the resulting simulation box was 201 Å × 204 Å × 181 Å. The simulation followed a similar protocol to that used for the AuNP in water. The only difference was that during the initial minimisation only the antibody atoms were kept immobile, while in the next steps, all atoms were free to move. To ensure the trajectories are representative from a statistical point of view, two independent 100 ns repetitions of the trajectory for each system were run. All trajectories were carefully analysed, and the general results matched.

### Root-mean-square distance (RMSD) and root-mean-square fluctuations (RMSF)

RMSD can be used to determine the structural variability of similar proteins or different conformations of the same protein^47^. Here the RMSD is defined as

1 $$RMSD(t)=\sqrt{\frac{{\sum }_{i=1}^{{N}_{atoms}}{\left|{\overrightarrow{r}}_{i}({t}_{1})-{\overrightarrow{r}}_{i}({t}_{2})\right|}^{2}}{{N}_{atoms}}}$$

where N_atoms_ is the number of atoms used in the analysis, and ${\overrightarrow{r}}_{i}$ (t) is the position of the ith atom at a given time t. In order to calculate the RMSD, the two structures to be compared are firstly considered as rigid bodies (with no internal flexibility allowed), then overlapped (aligned) using only translations and rotations. RMSDs were calculated using the backbone atoms only and were determined for the C_H3_ domains of the two heavy chains in each of the three simulations. Due to the insignificant differences in side-chain orientations, which could introduce additional noise, the side chains of residues are excluded from the RMSD calculation.

RMSF is an additional tool to track structural changes, where the RMSD is calculated for each residue. It is commonly known as “fluctuations” as it signifies each residue’s movement during the MD trajectory. RMSF reports an amplitude of residue fluctuation from the average position (in the aligned structures) over the trajectory analysed. Time average fluctuations of atoms of the same residue were calculated from the formula:

2 $${RMSF}_{k}=\sqrt{{\langle \frac{{\sum }_{i=1}^{{N}_{k}}{\left|{\overrightarrow{r}}_{i}(t)-{\langle {\overrightarrow{r}}_{i}\rangle }_{T}\right|}^{2}}{{N}_{k}}\rangle }_{T}}$$

where ${\overrightarrow{r}}_{i}(t)$ is the position of atom *i* in residue *k* at time *t*, *N*_*k*_ is the number of atoms in the residue and ${\langle {\overrightarrow{r}}_{i}\rangle }_{T}$ is the time average over the trajectory. Similar to RMSD, an additional component to the RMSF is used if two domains/chains change their relative orientations. To exclude these effects we amended our scripts to focus on each antibody fragment. The routinely used unit for RMSD and RMSF is Å (10^–10^ m), which is appropriate for the antibody length-scale.

### Supplementary Information

Supplementary Information.

## Data Availability

The datasets generated and/or analysed during the current study are available in this repository [10.15129/9d18b804-e053-4370-b535-5013e8ce1809].

## References

[1] Khan I, Saeed K, Khan I (2019). Nanoparticles: Properties, applications and toxicities. Arab. J. Chem..

[2] Farouq MAH, Al Qaraghuli MM, Kubiak-Ossowska K, Ferro VA, Mulheran PA (2021). Biomolecular interactions with nanoparticles: Applications for COVID-19. Curr. Opin. Colloid Interface Sci..

[3] Marycz K (2015). Application of gold nanoparticles of different concentrations to improve the therapeutic potential of autologous conditioned serum: Potential implications for equine regenerative medicine. J. Nanomater..

[4] Crescioli S (2022). Antibodies to watch in 2024. MAbs.

[5] Zinn S (2023). Advances in antibody-based therapy in oncology. Nat. Cancer.

[6] Ryle AP, Porter RR (1959). Parapepsins: Two proteolytic enzymes associated with porcine pepsin. Biochem. J..

[7] Porter RR (1959). The hydrolysis of rabbit y-globulin and antibodies with crystalline papain. Biochem. J..

[8] Wu TT, Kabat EA (1970). An analysis of the sequences of the variable regions of Bence Jones proteins and myeloma light chains and their implications for antibody complementarity. J. Exp. Med..

[9] Humphrey W, Dalke A, Schulten KVMD (1996). Visual molecular dynamics. J. Mol. Graph..

[10] Kubota T (2009). Engineered therapeutic antibodies with improved effector functions. Cancer Sci..

[11] Vidarsson G, Dekkers G, Rispens T (2014). IgG subclasses and allotypes: From structure to effector functions. Front. Immunol..

[12] Borrok MJ, Jung ST, Kang TH, Monzingo AF, Georgiou G (2012). Revisiting the role of glycosylation in the structure of human IgG Fc. ACS Chem. Biol..

[13] Kuo TT, Aveson VG (2011). Neonatal Fc receptor and IgG-based therapeutics. mAbs.

[14] Hall, T. A. BioEdit: A user-friendly biological sequence alignment editor and analysis program for Windows 95/98/NT. *Nucleic Acids Symp. Ser.* (1999).

[15] Chiu ML, Goulet DR, Teplyakov A, Gilliland GL (2019). Antibody structure and function: The basis for engineering therapeutics. Antibodies.

[16] Davis JH (2010). SEEDbodies: Fusion proteins based on strand-exchange engineered domain (SEED) CH3 heterodimers in an Fc analogue platform for asymmetric binders or immunofusions and bispecific antibodies. Protein Eng. Des. Sel. PEDS.

[17] Traxlmayr MW (2012). Construction of a stability landscape of the CH3 domain of human IgG1 by combining directed evolution with high throughput sequencing. J. Mol. Biol..

[18] Thies MJ (1999). Folding and association of the antibody domain CH3: Prolyl isomerization preceeds dimerization. J. Mol. Biol..

[19] Kabat EA, Wu TT, Foeller C, Perry HM, Gottesman KS (1992). Sequences of Proteins of Immunological Interest.

[20] Hasenhindl C (2013). Stability assessment on a library scale: A rapid method for the evaluation of the commutability and insertion of residues in C-terminal loops of the CH3 domains of IgG1-Fc. Protein Eng. Des. Sel. PEDS.

[21] Al Qaraghuli, M. M., Kubiak-Ossowska, K. & Mulheran, P. A. Thinking outside the laboratory: Analyses of antibody structure and dynamics within different solvent environments in molecular dynamics (MD) simulations. *Antibodies* **7**, (2018). 10.3390/antib7030021 PMC6640683 31544873

[22] Kubiak-Ossowska K, Jachimska B, Al Qaraghuli M, Mulheran PA (2019). Protein interactions with negatively charged inorganic surfaces. Curr. Opin. Colloid Interface Sci..

[23] al-Badri MA, Smith P, al-Jamal KT, Lorenz CD (2022). Nanomaterial functionalization modulates hard protein corona formation: Atomistic simulations applied to graphitic materials. Adv. Mater. Interfaces.

[24] Almagro JC (2004). Identification of differences in the specificity-determining residues of antibodies that recognize antigens of different size: Implications for the rational design of antibody repertoires. J. Mol. Recognit. JMR.

[25] McDonald IK, Thornton JM (1994). Satisfying hydrogen bonding potential in proteins. J. Mol. Biol..

[26] Raghunathan G, Smart J, Williams J, Almagro JC (2012). Antigen-binding site anatomy and somatic mutations in antibodies that recognize different types of antigens. J. Mol. Recognit. JMR.

[27] Hall, T. BioEdit: A user-friendly biological sequence alignment editor and analysis program for Windows 95/98/NT, 95–98 (1999).

[28] Gasteiger E, Walker JM (2005). Protein Identification and Analysis Tools on the ExPASy Server. The Proteomics Protocols Handbook.

[29] Sanner MF (1999). Python: A programming language for software integration and development. J. Mol. Graph. Model..

[30] Phillips JC (2005). Scalable molecular dynamics with NAMD. J. Comput. Chem..

[31] Martín-García F, Papaleo E, Gomez-Puertas P, Boomsma W, Lindorff-Larsen K (2015). Comparing molecular dynamics force fields in the essential subspace. PLoS ONE.

[32] Harris LJ, Larson SB, Hasel KW, McPherson A (1997). Refined structure of an intact IgG2a monoclonal antibody. Biochemistry.

[33] Dekkers G (2017). Affinity of human IgG subclasses to mouse Fc gamma receptors. MAbs.

[34] Shekhar S, Khan R, Khan AUR, Petersen FC (2019). Mouse IgG2a antibodies specific for the commensal streptococcus mitis show stronger cross-reactivity with streptococcus pneumoniae than IgG1 antibodies. J. Immunol. Res..

[35] Al Qaraghuli MM, Kubiak-Ossowska K, Ferro VA, Mulheran PA (2020). Antibody-protein binding and conformational changes: Identifying allosteric signalling pathways to engineer a better effector response. Sci. Rep..

[36] Heinz H, Vaia RA, Farmer BL, Naik RR (2008). Accurate simulation of surfaces and interfaces of face-centered cubic metals using 12–6 and 9–6 Lennard-Jones potentials. J. Phys. Chem. C.

[37] Cappabianca R, De Angelis P, Cardellini A, Chiavazzo E, Asinari P (2022). Assembling biocompatible polymers on gold nanoparticles: Toward a rational design of particle shape by molecular dynamics. ACS Omega.

[38] Kalčec N (2022). Transformation of L-DOPA and dopamine on the surface of gold nanoparticles: An NMR and computational study. Inorg. Chem..

[39] Zhu C, Hoff SE, Hémadi M, Heinz H (2023). Accurate and ultrafast simulation of molecular recognition and assembly on metal surfaces in four dimensions. ACS Nano.

[40] Azman N, Nguyen TX, Kah JCY (2021). Dynamics of human serum albumin corona formation on gold nanorods with different surface ligands in silico. J. Phys. Chem. B.

[41] Flint Z (2024). Mechanistic insights behind the self-assembly of human insulin under the influence of surface-engineered gold nanoparticles. ACS Chem. Neurosci..

[42] Guterres H (2022). CHARMM-GUI high-throughput simulator for efficient evaluation of protein–ligand interactions with different force fields. Protein Sci..

[43] Tiwari V, Garg S, Karmakar T (2024). Insights into the interactions of peptides with monolayer-protected metal nanoclusters. ACS Appl. Bio Mater..

[44] Touzeau J (2021). Theoretical and experimental elucidation of the adsorption process of a bioinspired peptide on mineral surfaces. Langmuir.

[45] Riccardi L (2021). Molecular recognition by gold nanoparticle-based receptors as defined through surface morphology and pockets fingerprint. J. Phys. Chem. Lett..

[46] Essmann U (1995). A smooth particle mesh Ewald method. J. Chem. Phys..

[47] Carugo O, Pongor S (2001). A normalized root-mean-square distance for comparing protein three-dimensional structures. Protein Sci. Publ. Protein Soc..

