# Supplementary material for: Exploiting the Fc base of IgG antibodies to create functional nanoparticle conjugates
Source: Sci Rep. 2024 Jun 27;14:14832. doi: 10.1038/s41598-024-65822-7 (PMC11211340; doi:10.1038/s41598-024-65822-7)
Supplement: Supplementary file 1 — Supplementary Information. [file 41598_2024_65822_MOESM1_ESM.pdf]

Supplementary Material

Figure S1: Sequences alignment of the 54 crystal structures CH3 domains.

```
                                                10        20        30        40        50        60        70        80        90       100
                                       ....|....|....|....|....|....|....|....|....|....|....|....|....|....|....|....|....|....|....|....|
1DN2:A|PDBID|CHAIN|SEQUENCE            GQPREPQVYTLPPSREEMTKNQVSLTCLVKGFYPSDIAVEWESNGQPENNYKTTPPVLDSDGSFFLYSKLTVDKSRWQQGNVFSCSVMHEALHNHYTQKS
1E4K:A|PDBID|CHAIN|SEQUENCE            ....................................................................................................
1FC1:A|PDBID|CHAIN|SEQUENCE            ....................................................................................................
1FC2:D|PDBID|CHAIN|SEQUENCE            ....................................................................................................
1H3T:A|PDBID|CHAIN|SEQUENCE            ....................................................................................................
1H3U:A|PDBID|CHAIN|SEQUENCE            ....................................................................................................
1H3V:A|PDBID|CHAIN|SEQUENCE            ....................................................................................................
1H3X:A|PDBID|CHAIN|SEQUENCE            ....................................................................................................
1H3Y:A|PDBID|CHAIN|SEQUENCE            ....................................................................................................
1T89:A|PDBID|CHAIN|SEQUENCE            ....................................................................................................
2GJ7:A|PDBID|CHAIN|SEQUENCE            ....................................................................................................
3DNK:A|PDBID|CHAIN|SEQUENCE            ....................................................................................................
3DO3:A|PDBID|CHAIN|SEQUENCE            ....................................................................................................
3RY6:A|PDBID|CHAIN|SEQUENCE            ....................................................................................................
1T83:A|PDBID|CHAIN|SEQUENCE            ....................................................................................................
4BYH:A|PDBID|CHAIN|SEQUENCE            ....................................................................................................
4NHG:Y|PDBID|CHAIN|SEQUENCE            ....................................................................................................
4NHH:A|PDBID|CHAIN|SEQUENCE            ....................................................................................................
1HZH:H|PDBID|CHAIN|SEQUENCE            ...............D.L..................................................................................
1OQO:A|PDBID|CHAIN|SEQUENCE            ...............D.L..................................................................................
1OQX:A|PDBID|CHAIN|SEQUENCE            ...............D.L..................................................................................
2IWG:A|PDBID|CHAIN|SEQUENCE            ...............D.L..................................................................................
2WAH:A|PDBID|CHAIN|SEQUENCE            ...............D.L..................................A...............................................
3AVE:A|PDBID|CHAIN|SEQUENCE            ...............D.L..................................................................................
3AY4:A|PDBID|CHAIN|SEQUENCE            ...............D.L..................................................................................
3D6G:A|PDBID|CHAIN|SEQUENCE            ...............D.L..................................................................................
3S7G:D|PDBID|CHAIN|SEQUENCE            ...............D.L..................................................................................
3SGJ:A|PDBID|CHAIN|SEQUENCE            ...............D.L..................................................................................
3SGK:A|PDBID|CHAIN|SEQUENCE            ...............D.L..................................................................................
3WJJ:A|PDBID|CHAIN|SEQUENCE            ...............D.L..................................................................................
3WJL:A|PDBID|CHAIN|SEQUENCE            ...............D.L..................................................................................
4ZNE:J|PDBID|CHAIN|SEQUENCE            ...............D.L..................................................................................
1L6X:A|PDBID|CHAIN|SEQUENCE            ...............D.L..................................................................................
2DTS:A|PDBID|CHAIN|SEQUENCE            ...............D.L..................................................................................
4WI2:A|PDBID|CHAIN|SEQUENCE            ...............D.L..................................................................................
2J6E:A|PDBID|CHAIN|SEQUENCE            ...............D.L..................................................................................
4ACP:A|PDBID|CHAIN|SEQUENCE            ...............D.L..................................A...............................................
4B7I:A|PDBID|CHAIN|SEQUENCE            ...............D.L..................................A...............................................
4HAF:A|PDBID|CHAIN|SEQUENCE            ........................................................M...........................................
4HAG:A|PDBID|CHAIN|SEQUENCE            ........................................................M...........................................
4L4J:A|PDBID|CHAIN|SEQUENCE            ........................................................M...........................................
4WWI:F|PDBID|CHAIN|SEQUENCE            ...........................................S.......N....M........................I..................
4ZNC:F|PDBID|CHAIN|SEQUENCE            ...........................................S.......N....M........................I..................
1ADQ:A|PDBID|CHAIN|SEQUENCE            ..............Q.....................................................R.........E.....................
4C54:A|PDBID|CHAIN|SEQUENCE            ..............Q.....................................................R.........E.....................
4C55:A|PDBID|CHAIN|SEQUENCE            ..............Q.....................................................R.........E.....................
4D2N:A|PDBID|CHAIN|SEQUENCE            ..............Q.....................................................R.........E.....................
4B53:A|PDBID|CHAIN|SEQUENCE            -E............Q.....................................................R.........E.....................
3HKF:A|PDBID|CHAIN|SEQUENCE            .R.KA.....I..PK.Q.A.DK.....MITD.F.E..T...QW....AE...N.Q.IM.T...Y.V....N.Q..N.EA..T.T...L..G....H.E..
1IGY:D|PDBID|CHAIN|SEQUENCE            .K..A.....I..PK.Q.A.DK.....MITD.F.E..T...Q.D..APE...N.Q.IM.T...Y.V....N.Q..N.EA..T.T...L..G....H.E..
2RGS:A|PDBID|CHAIN|SEQUENCE            .LV.A....I...PA.QLSRKD.......V..N.G..S...T...HT.E...D.A........Y.I....NMKT.K.EKTDS...N.R..G.K.Y.LK.T
1IGT:D|PDBID|CHAIN|SEQUENCE            .SV.A....V...PE.....K..T...M.TD.M.E..Y...TN..KT.L...N.E........Y.M....R.E.KN.VER.SY....V..G....H.T..
2VUO:A|PDBID|CHAIN|SEQUENCE            ...L..K...MG.P...LSSRS.....MIN.......S....K..KA.D......A.......Y......S.PT.E..R.D..T................
1I1C:A|PDBID|CHAIN|SEQUENCE            .T..G.....MA.PK....QS...I..M......P..YT..KM....QE...N...TM.T...Y......N.K.ET.....T.T...L..G.E.EH.E..

                                       ....|....
1DN2:A|PDBID|CHAIN|SEQUENCE            LSL
1E4K:A|PDBID|CHAIN|SEQUENCE            ...SPGK
1FC1:A|PDBID|CHAIN|SEQUENCE            ...SPG
1FC2:D|PDBID|CHAIN|SEQUENCE            ...SPG
1H3T:A|PDBID|CHAIN|SEQUENCE            ...SPGK
1H3U:A|PDBID|CHAIN|SEQUENCE            ...SPGK
1H3V:A|PDBID|CHAIN|SEQUENCE            ...SPGK
1H3X:A|PDBID|CHAIN|SEQUENCE            ...SPGK
1H3Y:A|PDBID|CHAIN|SEQUENCE            ...SPGK
1T89:A|PDBID|CHAIN|SEQUENCE            ...SPGK
2GJ7:A|PDBID|CHAIN|SEQUENCE            ...SPGK
3DNK:A|PDBID|CHAIN|SEQUENCE            ...SPGK
3DO3:A|PDBID|CHAIN|SEQUENCE            ...SPGK
3RY6:A|PDBID|CHAIN|SEQUENCE            ...S
1T83:A|PDBID|CHAIN|SEQUENCE            ...SPGK
4BYH:A|PDBID|CHAIN|SEQUENCE            ...SPGTK
4NHG:Y|PDBID|CHAIN|SEQUENCE            ...SPGK
4NHH:A|PDBID|CHAIN|SEQUENCE            ...SPGK
1HZH:H|PDBID|CHAIN|SEQUENCE            ...SPGK
1OQO:A|PDBID|CHAIN|SEQUENCE            ...SPGK
1OQX:A|PDBID|CHAIN|SEQUENCE            ...SPGK
2IWG:A|PDBID|CHAIN|SEQUENCE            ...
2WAH:A|PDBID|CHAIN|SEQUENCE            ...SP
3AVE:A|PDBID|CHAIN|SEQUENCE            ...SPGK
3AY4:A|PDBID|CHAIN|SEQUENCE            ...SPGK
3D6G:A|PDBID|CHAIN|SEQUENCE            ...SP
3S7G:D|PDBID|CHAIN|SEQUENCE            ...SPGK
3SGJ:A|PDBID|CHAIN|SEQUENCE            ...SPGK
3SGK:A|PDBID|CHAIN|SEQUENCE            ...SPGK
3WJJ:A|PDBID|CHAIN|SEQUENCE            ...SP
3WJL:A|PDBID|CHAIN|SEQUENCE            ...SP
4ZNE:J|PDBID|CHAIN|SEQUENCE            ...SPGK
1L6X:A|PDBID|CHAIN|SEQUENCE            ...
2DTS:A|PDBID|CHAIN|SEQUENCE            ...SPGK
4WI2:A|PDBID|CHAIN|SEQUENCE            ...S
2J6E:A|PDBID|CHAIN|SEQUENCE            ...SPGK
4ACP:A|PDBID|CHAIN|SEQUENCE            ...SPGGTK
4B7I:A|PDBID|CHAIN|SEQUENCE            ...
4HAF:A|PDBID|CHAIN|SEQUENCE            ...SPGK
4HAG:A|PDBID|CHAIN|SEQUENCE            ...SPGK
4L4J:A|PDBID|CHAIN|SEQUENCE            ...SPGK
4WWI:F|PDBID|CHAIN|SEQUENCE            ...SPGK
4ZNC:F|PDBID|CHAIN|SEQUENCE            ...SPGK
1ADQ:A|PDBID|CHAIN|SEQUENCE            ...
```

```
4C54:A|PDBID|CHAIN|SEQUENCE  ...SLGK
4C55:A|PDBID|CHAIN|SEQUENCE  ...SLGK
4D2N:A|PDBID|CHAIN|SEQUENCE  ...SLGK
4B53:A|PDBID|CHAIN|SEQUENCE  ...SLGK
3HKF:A|PDBID|CHAIN|SEQUENCE  ..HSPGK
1IGY:D|PDBID|CHAIN|SEQUENCE  ..H
2RGS:A|PDBID|CHAIN|SEQUENCE  I.RSPG
1IGT:D|PDBID|CHAIN|SEQUENCE  F.R
2VUO:A|PDBID|CHAIN|SEQUENCE  I.RSPGK
1I1C:A|PDBID|CHAIN|SEQUENCE  ..HSPGK
```

Table S1: Electrostatic parameters of the C$_{H3}$ domains

Summary of the calculated PI, Mwt, and the total electrostatic potential of the analysed crystal structures. The total electrostatic energy was measured for the CH3 domains, as shown in Figure 3.

Crystal structure	PI	Mwt (kDa)
**1IGT (Mouse IgG2a)**	6.08	12.005
**2RGS (Mouse IgG2b)**	7.94	11.916
**1IGY (Mouse IgG1)**	5.64	11.656
**1I1C (Rat IgG2A)**	5.89	12.179
**2VUO (Rabbit (IgG)**	5.88	12.022
**3DO3 (Human IgG1)**	6.31	12.109
**4HAF (Human IgG2)**	6.31	12.141
**4WWI (Human IgG3)**	5.86	12.114
**4C55 (Human IgG4)**	5.52	12.126

Figure S2: Surface-mapped electrostatic potential of the nine selected structures

The Fc base charges were analysed for the nine selected structures oriented in similar configuration to Figure 3. Measurements were calculated utilising Python Molecule Viewer (PMV) Version 1.5.6. The produced energies were mapped to the surface with medium surface quality and 1 Å distance from the surface. The map colours were coded as white: neutral, blue: positively charged, red: negatively charged. PDB entries are given at the lower corner of each image.

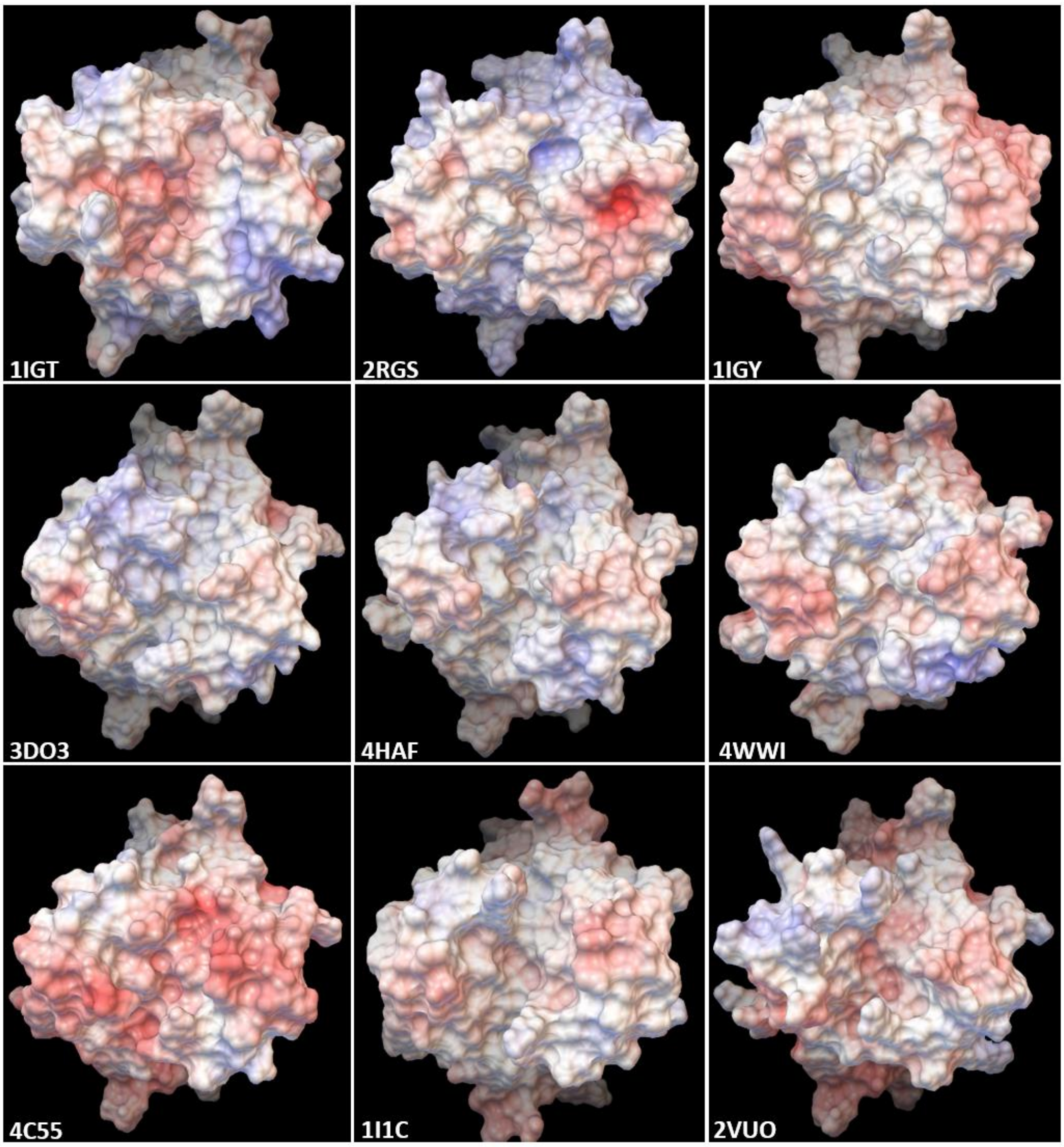

Figure S3: The time evolution of the minimum separations (in Å) between the interacting residues of the I1GT chain and the nanoparticle. (a) chain D and charged AuNP; (b) chain B and uncharged AuNP; (c) chain D and uncharged AuNP. The residue numbers are shown in the legend.

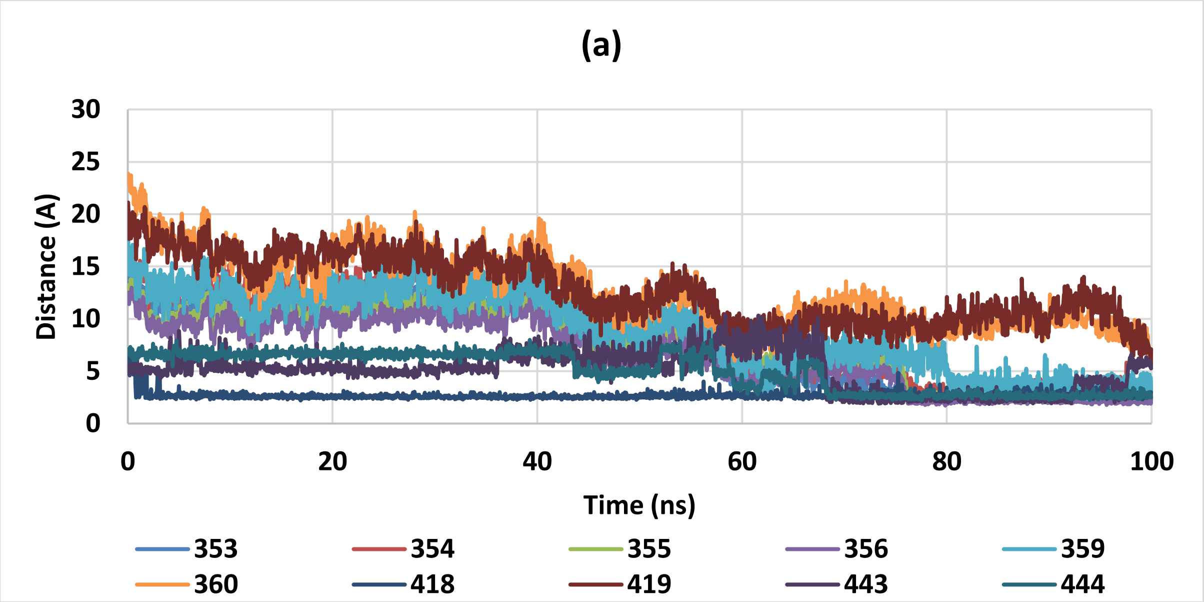

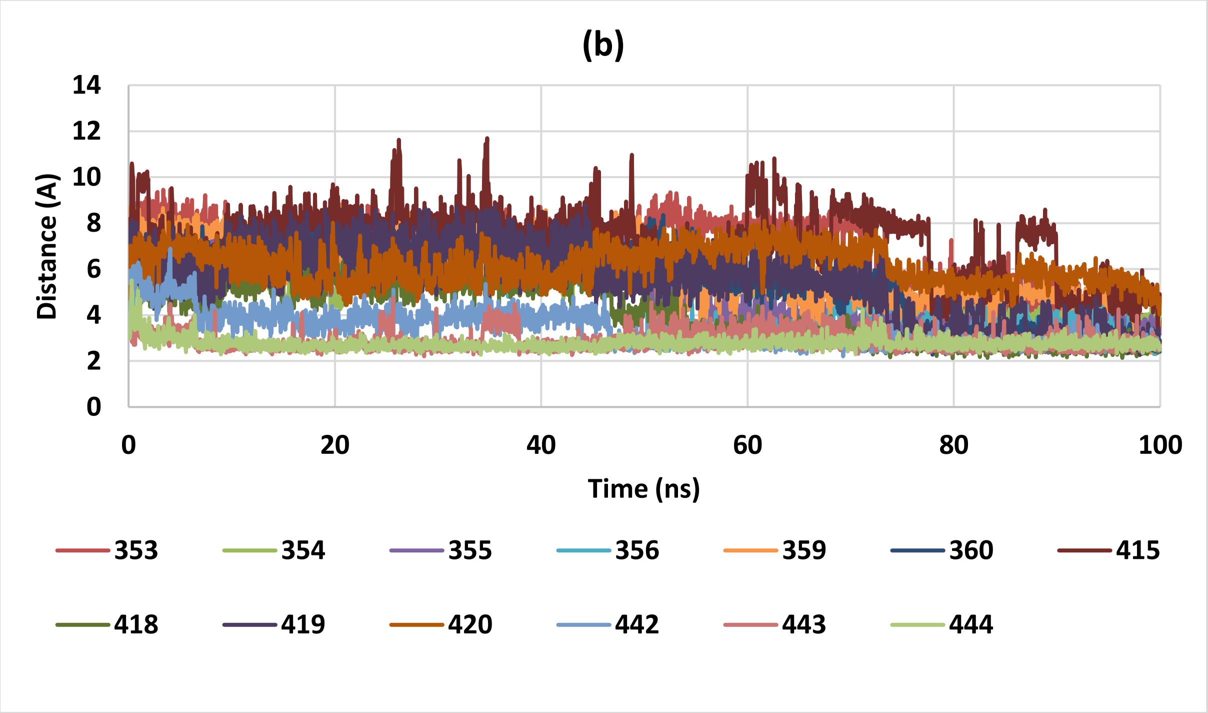

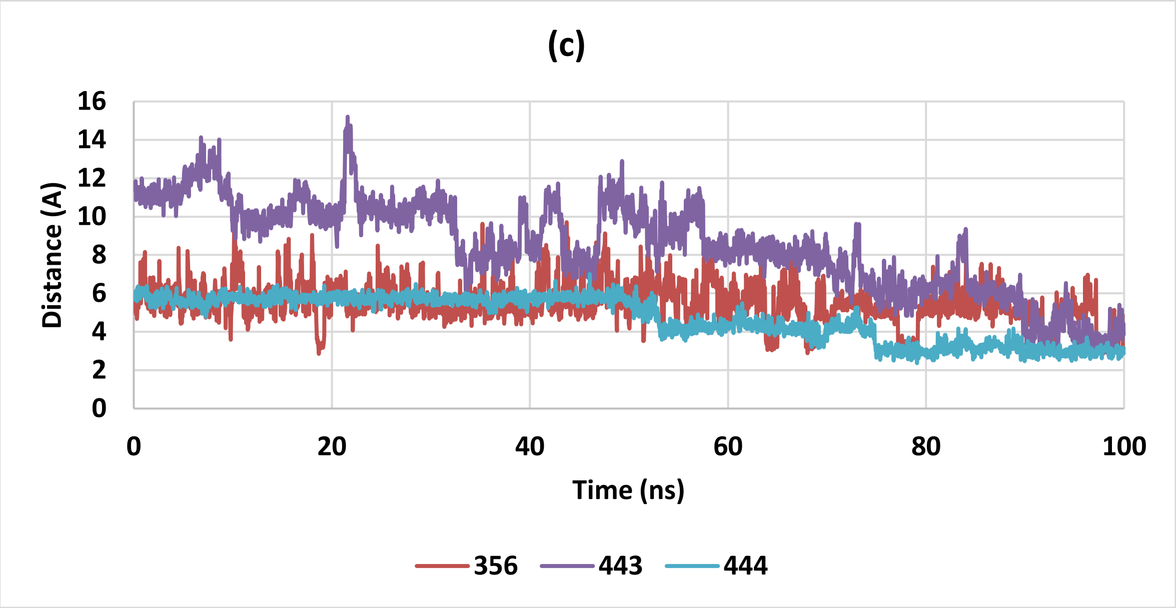
(c)
Distance (A)
16
14
12
10
8
6
4
2
0
0
20
40
60
80
100
Time (ns)
356
443
444

Figure S4: RMSF analysis of the four antibody chains.

RMSF values for the residues in (a) Chain B and (b) Chain D from the C_{H3} domains of I1GT simulations with and without nanoparticles.

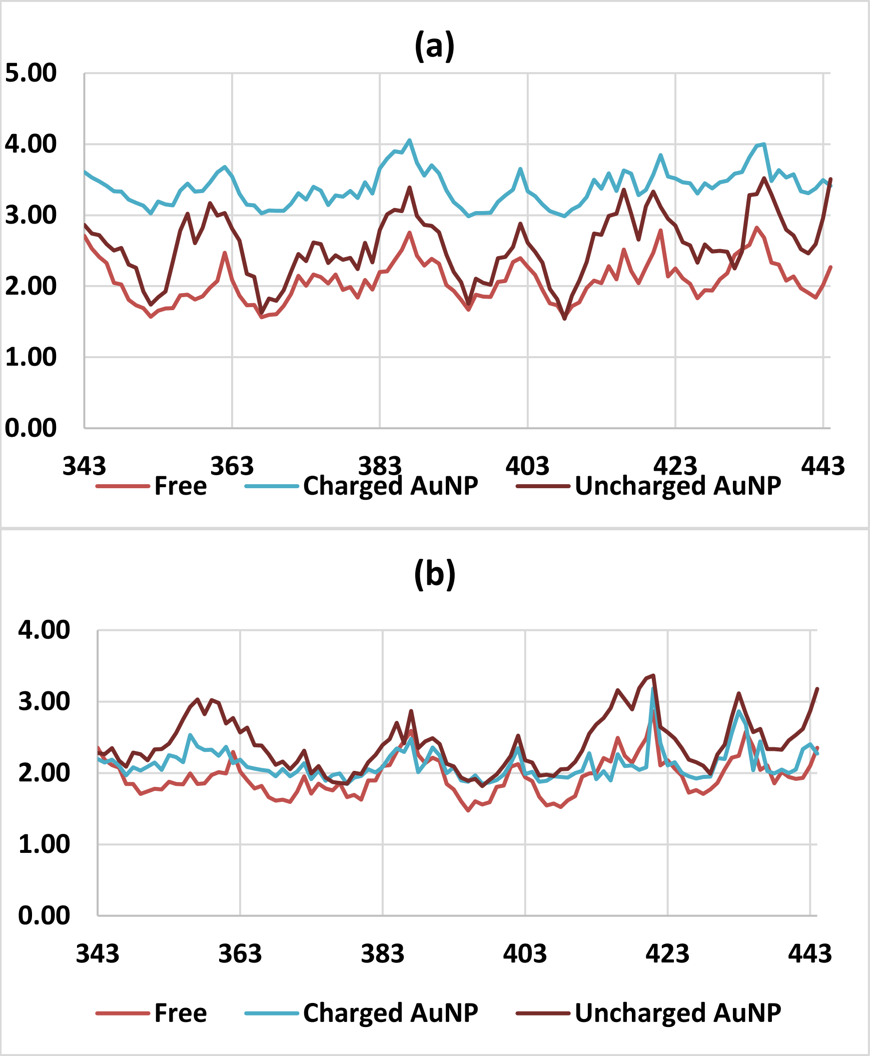

Table S2: RMSD comparisons for the C_{H3} domain

The RMSD values (in Å) were compared by recording the average values of the last 50ns of each of the three simulations. The standard error on the means is also given.

	1IGT (chain B)	1IGT-charged 2.5nm AuNP (chain B)	1IGT-uncharged 2.5nm AuNP (chain B)	1IGT (chain D)	1IGT-charged 2.5nm AuNP (chain D)	1IGT-uncharged 2.5nm AuNP (chain D)
**Average**	1.79	1.54	1.53	1.15	1.56	1.96
**Standard Deviation**	0.14	0.15	0.16	0.08	0.26	0.12
**SEM**	0.003	0.004	0.004	0.002	0.007	0.003
